# Risk factors for UK *Plasmodium falciparum* cases

**DOI:** 10.1186/1475-2875-13-298

**Published:** 2014-08-04

**Authors:** Amy Pinsent, Jonathan M Read, Jamie T Griffin, Valerie Smith, Peter W Gething, Azra C Ghani, Geoffrey Pasvol, T Déirdre Hollingsworth

**Affiliations:** 1 Department of Infectious Disease Epidemiology, MRC Centre for Outbreak Analysis and Modelling, School of Public Health, Faculty of Medicine, Imperial College London, St Mary’s Campus, Norfolk Place, London W2 1PG, UK; 2 Department of Epidemiology and Population Health, Faculty of Health and Life Sciences, Institute of Infection and Global Health, University of Liverpool, Liverpool, UK; 3 NIHR Health Protection Research Unit in Emerging and Zoonotic Infections, Liverpool L69 7BE, UK; 4 Public Health England, Malaria Reference Laboratory, London School of Hygiene & Tropical Medicine, Keppel St, London WC1E 7HT, UK; 5 Department of Zoology, Spatial Ecology and Epidemiology Group, Tinbergen Building, University of Oxford, South Parks Road, Oxford, UK; 6 Department of Infection & Tropical Medicine, Imperial College London, Lister Unit, Northwick Park Hospital, Middlesex HA1 3UJ, UK; 7 Warwick Mathematics Institute, University of Warwick, Coventry CV4 7AL, UK; 8 School of Life Sciences, University of Warwick, Coventry CV4 7AL, UK; 9 Department of Clinical Sciences, Liverpool School of Tropical Medicine, Pembroke Place, Liverpool L3 5QA, UK

**Keywords:** Imported malaria, Falciparum, Travel

## Abstract

**Background:**

An increasing proportion of malaria cases diagnosed in UK residents with a history of travel to malaria endemic areas are due to *Plasmodium falciparum*.

**Methods:**

In order to identify travellers at most risk of acquiring malaria a proportional hazards model was used to estimate the risk of acquiring malaria stratified by purpose of travel and age whilst adjusting for entomological inoculation rate (EIR) and duration of stay in endemic countries.

**Results:**

Travellers visiting friends and relatives and business travellers were found to have significantly higher hazard of acquiring malaria (adjusted hazard ratio (HR) relative to that of holiday makers 7.4, 95% CI 6.4–8.5, p < 0. 0001 and HR 3.4, 95% CI 2.9-3.8, p < 0. 0001, respectively). All age-groups were at lower risk than children aged 0–15 years.

**Conclusions:**

These estimates of the increased risk for business travellers and those visiting friends and relatives should be used to inform programmes to improve awareness of the risks of malaria when travelling.

## Background

The majority of travellers to malaria endemic areas (~6.5million per year from the UK [1]) do not contract malaria due to the uptake of preventative and personal protective measures such as malaria chemoprophylaxis, bed nets and insect repellent. Nevertheless, malaria acquired in endemic regions and imported into non-endemic countries accounts for a considerable and largely preventable burden of morbidity and mortality throughout Europe [2,3]. The UK has one of the highest rates of imported malaria among non-endemic countries, with around 2,000 cases per year resulting in five to 16 deaths [4]. Whilst there has been a decrease in imported malaria cases in the UK since 2000, the UK saw an increase in the number of cases imported in 2010 and 2011 [5]. Over this period there has also been an increase in the proportion of cases attributable to *Plasmodium falciparum* rather than *Plasmodium vivax*[3]*. Plasmodium falciparum* is associated with severe symptoms and mortality [6-8]. It is, therefore, important to identify groups at high risk of contracting malaria to effectively target preventative health advice. Developing reliable measures of risk in the wider travelling population is also necessary to inform proposals to reduce levels of chemoprophylaxis amongst those travelling to low risk areas [9,10]. The work presented here focuses on *P. falciparum* in 2007, not only because of to its serious consequences, but because of the availability of detailed global estimates of *P. falciparum* parasite prevalence in 2007 [11,12] and a model which can be used to convert these estimates to the entomological inoculation rate (EIR) [13].

A large proportion of UK malaria cases are acquired whilst travelling to visit friends and relatives (hereafter referred to as VFR) [14,15]. The majority of these infections are acquired in West Africa [16], even though these areas are not frequent destinations for UK residents [3,14]. Travellers VFR visit areas where malaria is more readily transmitted and tend to stay for longer than those who travel for any other purpose [17-20]. An increase in risk of infection beyond that due to increased exposure and longer duration of travel is supported by the observation that travellers VFR have often lived in malarial areas previously and may assume that they are immune, even though their residual level of immunity may not be sufficient to prevent infection [18-23]. Studies have also suggested that individual concerns about health care services and side-effects of chemoprophylaxis present themselves as barriers to uptake of chemoprophylaxis in VFR travellers [24]. This perception of immunity and distrust of health services may help to explain poor uptake of prophylaxis and pre-travel health advice amongst travellers VFR [3,25] as well as the large number of cases in this group.

A number of imported cases are in children (10% under the age of 15 in 2007), suggesting that they may be at higher risk of acquiring malaria as travellers [7]. Children are believed to be at greater risk of acquiring malaria in general and children <6 years of age have a significantly higher risk of presenting with clinical malaria [7,26]. In addition, adequate uptake of, and compliance with, prophylaxis by children may be poor, particularly when travelling with family members for the purposes of visiting friends and relatives [21,22,26-28]

The risk of acquiring malaria whilst visiting a malaria endemic country is multi-factorial. Longer duration of stay and higher transmission rates in the destination country increase the probability of acquisition, as does poor adherence to preventative measures. Most previous analyses have highlighted this, but the majority have been unable to disentangle these different effects. A more recent study characterized transmission risk in the destination country by malaria incidence and found that this did not correlate well with the risk of travellers acquiring malaria [9]. When this model was extended to account for duration of stay in high risk areas, it was shown that chemoprophylaxis of travellers may only be cost effective in moderate to high incidence areas [29]. In this study a more direct measure of exposure, the entomological inoculation rate (EIR), or the number of infectious bites per person per year, calculated from parasite prevalence estimates for 2007 [11,12], is used to estimate the risk of acquiring malaria in different groups of travellers.

## Methods

### Data sources

#### UK cases of Plasmodium falciparum in 2007

The Malaria Reference Laboratory (MRL) of the UK Health Protection Agency (HPA) obtained detailed passive surveillance reports on cases of malaria (confirmed by blood films or tissue histology) from clinicians and laboratories in the UK [3]. The notifying laboratory and clinician provided data on exposure risk for each case, including their country of usual residence (excluding non-UK residents), the reason for travel, the country or region visited and duration of travel. Whilst there is likely to be underreporting of cases of malaria in the UK, these data give the most comprehensive picture of imported malaria in the UK.

#### UK travellers to malaria endemic areas

The travel patterns of UK residents were obtained from two sources. Firstly, the number of visitors to each country was collated from World Tourism Organization (WTO) data from member states on the number of visitors to each country by country of residence [1]. Data from 2007 was used if available, or for the closest year for which data were available, and online sources of information for the few countries for which no data were available.

Secondly, data on the purpose of travel and the age of travellers to malaria endemic areas were obtained from the TravelPac, provided by the Office for National Statistics [30]. These data are collected as part of the International Passenger Survey (IPS) in which personal interviews are conducted with approximately 250,000 randomly selected travellers annually (~0.2% of the total). The data are stratified by country unless the sample size is too small in which case data are aggregated across countries. Since only a small proportion of UK travellers visit malaria endemic areas, the data in this survey on the proportion of travellers visiting for particular reasons or within particular age-groups has to be aggregated across several malaria endemic countries. For example, since “Other Africa” included 43 African countries, the proportion of the UK visitors aged 0–15 years of age who visited friends and relatives was the same across a number of African countries. To overcome this limitation in the data, searches for other data sources were carried out, but extensive internet searches only found data on reason for travel from an extremely limited number of countries and the data was never stratified for travellers from the UK (Additional file 1). Therefore this analysis uses the systematic data collected by the IPS, despite its clear limitations, since it is the only source for data systematically captured from UK travellers. To investigate the impact of this assumption the analysis was repeated for just Uganda, which had the most suitable travel data.

#### Within-country transmission rate

Malaria transmission is highly heterogeneous at all scales, from continent to village [12,31,32]. Since the destination of travel for malaria cases is only reported at the country level, the exposure risk (EIR) was estimated as an average at the country level. *P. falciparum* parasite prevalence estimates in 2–10 year olds were transformed to population-weighted national-level mean EIR estimates by first using the relationship derived from fitting of a mathematical model to data on the EIR to prevalence amongst 2–10 year olds relationship from 34 locations across Africa [13] and then calculating the population-weighted mean (see Additional file 2 and [11,12]). In order to investigate sensitivity to this assumption of population weighting the analysis was repeated with the EIR average by area within a country.

### Statistical analysis

#### Crude incidence

To demonstrate general trends in the data the incidence of imported malaria to the UK in 2007 was calculated for three key variables of interest: reason for travel, age group and EIR (depending on destination visited). Incidence was calculated as number of cases imported by each group of interest divided by the total number of visitors to all malaria endemic areas (MEAs) in that group as provided in the WTO data for country and IPS data for reason for travel and age. Binomial confidence intervals were calculated. This analysis does not account for missing data, and therefore a more sophisticated model was used for the full analysis.

#### Statistical model

In order to assess the relative hazards faced by different travellers, it was assumed that the risk of acquiring malaria depends on the daily level of exposure (the EIR) and the duration of stay in a malarial endemic country. As such, it was assumed that the probability of infection per bite is the same for all individuals in all transmission settings. A log-linear model was used to calculate the hazard of acquiring malaria in which the time of infection was assumed to have occurred at any point during the reported visit to a malaria endemic area and the daily hazard of infection is determined by the EIR (assuming a constant rate and thus ignoring both any nonlinear relationship between EIR and force of infection and any seasonality in transmission since data on the months of visits was not available). Two covariates were considered both in univariable and multivariable models – the age of the participant (as a categorical variable) and the reported reason for travel (a detailed description of the model and model equations are provided in Additional file 3). The hazard was calculated as an aggregate measure of the effect of the use of preventative measures, such as bed nets, insect repellent and chemoprophylaxis, because detailed usage statistics were not available for travellers in general.

Since one or more of the covariates or the destination of travel were missing for a proportion of cases (235 (23%) missing information on destination, 455 (45%) missing reason for travel and 4 (<1%) missing age), parameter estimation was performed using an expectation-maximization algorithm [33]. A detailed description of the method is provided in Additional file 3.

## Results

**Figure 1:**
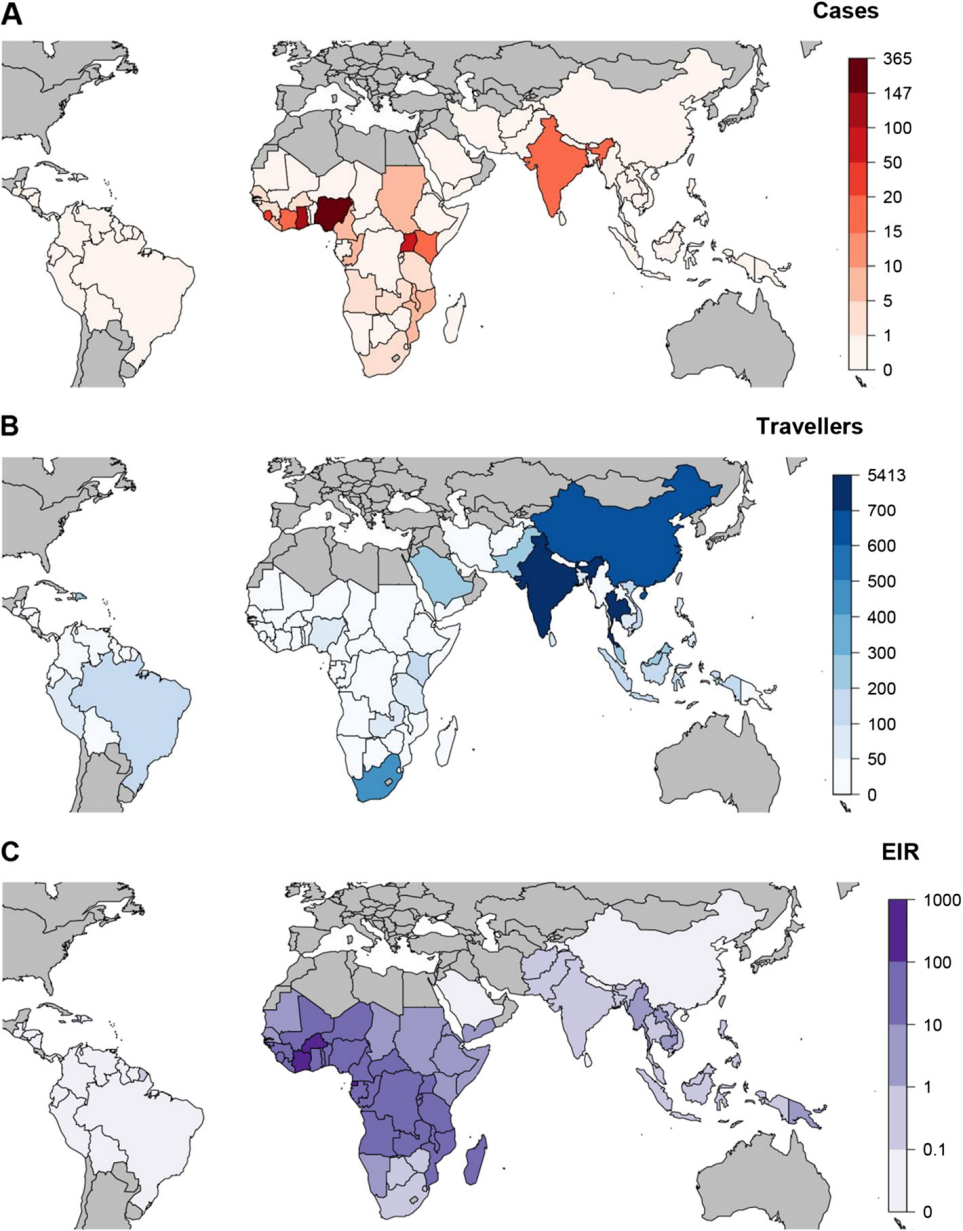
**Maps of the country of origin of *P. falciparum *cases amongst UK residents travelling to malaria endemic areas in 2007, number of UK residents travelling to each malaria-endemic country and the estimated entomological infection rate for each malaria-endemic country. (A)** The number of cases imported to the UK in 2007 by country of reported travel exposure. **(B)** The number of UK residents travelling to malaria-endemic areas in 2007 [1]. White areas are those that are considered non-endemic. **(C)** Estimated entomological infection rate (EIR, in units of infectious bites per person per year) for each malaria-endemic country.

One thousand, one hundred and fifty four cases of *P. falciparum* were reported by the malaria reference laboratory in 2007. Excluding those who were not UK residents, whose country of residence was unknown and those whose purpose of travel were foreign visitor, a new entrant to the UK or foreign students, resulted in 1004 cases. Of these, 11 were mixed infections: three *P. falciparum*/*P. vivax*, four *P. falciparum*/*Plasmodium malariae* and four *P. falciparum/Plasmodium ovale.* In those cases for whom the country of travel was reported (796/1004, 79%), the most frequent country where the cases had contracted malaria were Nigeria (365 cases, 46%), Ghana (147 cases, 18%) and Uganda (54 cases, 7%) (Figure 1A). Travellers from the UK visit a number of malaria endemic countries (Figure 1B), and their destinations reflect a large range of EIR (Figure 1C). For those malaria cases that reported their reason for travel (573/1,004, 57%), the most common reason was travelling to visit friends and relatives (VFR), Table 1. Most cases were reported amongst adults aged 24 to 55 years of age, although there were a substantial number of children (Table 1).

**Table 1:** Characteristics of cases of imported malaria in the UK in 2007 and crude incidence for each group

	**Cases (%)**	**Travellers 1,000 s (%)**[30]	Duration of visit (days) Median (IQR*)	**Crude incidence per 10,000**
**Purpose**				
VFR	450 (82%)	1,976 (30%)	23 (14–40)	2. 3 (2. 1–2. 5)
Misc*	6 (1. 3%)	282 (4%)	300 (14–365)	0. 21 (0. 1–0. 4)
Business	35 (6. 4%)	774 (12%)	30 (16–89)	0. 45 (0. 31–0. 61)
Holiday	58 (11%)	3,638 (55%)	19 (14–42)	0. 15 (0. 12–0. 20)
Missing	455		30 (19–42)	
**Age group (years)**				
0–15	97 (9. 7%)	302 (4. 5%)	30 (21–85)	3. 2 (2. 6–3. 9)
16–24	102 (10%)	611 (9%)	30 (17. 5–51)	1. 7 (1. 4–2. 0)
25–34	198 (20%)	1,520 (23%)	25 (14–42)	1. 3 (1. 1–1. 5)
35–44	270 (27%)	1,424 (21%)	21 (14–32)	1. 9 (1. 7–2. 1)
45–54	203 (20%)	1,345 (20%)	21 (14–30)	1. 5 (1. 3–1. 7)
55–64	83 (8. 3%)	973 (14. 5%)	30 (15–55. 5)	0. 85 (0. 68 – 1. 04)
65+	47 (4. 7%)	495 (8%)	58. 5 (30–116)	0. 95 (0. 70–1. 23)
Missing	4			
**Total**	1,004	6,670		

*Misc: Miscellaneous purpose of travel. IQR: Inter-quartile range.

**Figure 2:**
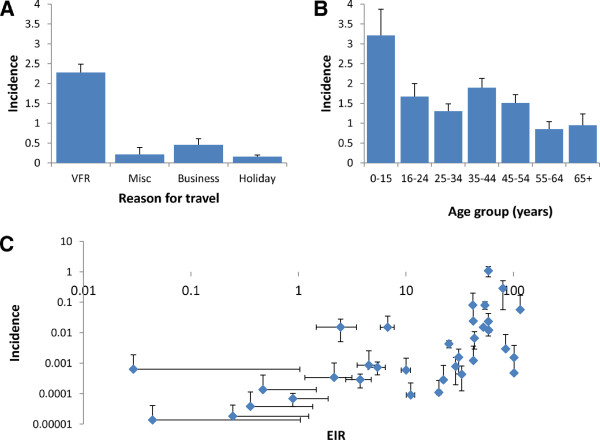
Crude incidence per 10,000 travellers stratified by (A) reason for travel, (B) age group and (C) EIR in country of travel.

Across all traveller groups to MEAs VFR travellers had the highest crude incidence of malaria cases at 2.3 cases per 10,000 travellers (95% CI 2.1-2.5) (Figure 2, Table 1). Business travellers also had a high level of crude incidence, with 0.45 cases per 10,000 travellers (95% CI 0.31-0.61). When considering age alone, children aged 0–15 years had the highest incidence (3.2, 95% CI (2.6-3.9), while adults aged 55–64 years had the lowest incidence of all age groups (0.95 cases per 10,000 travellers (95% CI 0.70-1.23)). There was increasing incidence with increasing EIR (Figure 2C).

Using the full statistical model which accounted for missing data, the univariable analyses showed that including purpose of travel and age significantly improved the fit of the model (*p <* 0.0001, Table 2). The parameter estimates were similar for both the univariable and multivariable models (including both reason for travel (p < 0.0001) and age (p < 0.0001)), as such, the results from the multivariable model are focused on. Both travellers visiting friends and relatives (adjusted HR 7.4, 95% confidence interval (CI) 6.4-8.5, p < 0.0001) and business travellers (adjusted HR 3.4, 95% CI 2.9-3.8, p < 0.0001) were at significantly higher risk compared to holiday-makers (Table 2). When the univariable analysis was repeated without missing data, using the 438 of 1,004 cases in the database the hazard ratios had a similar qualitative pattern, but with lower magnitude of the increased risk for travellers visiting friends and relatives (adjusted HR 2.5 (2.3-2.7), p < 0.0001) and business travellers (adjusted HR 1.6 (1.4-1.7), p < 0. 0001). Those travelling for miscellaneous reasons remained at lower risk (adjusted HR 0.87 (0.80–0.94), p = 0.03) compared to holiday-makers.

**Table 2:** Estimated hazard ratio (univariable model) and adjusted hazard ratio (multivariable model) for acquiring malaria conditional on the estimated level of exposure in the destination country

	**Hazard Ratio (95% confidence interval)**	***p***	**Adjusted hazard ratio (95% confidence interval)**	***p***
**Purpose**		<0. 0001*		<0. 0001*
VFR	6. 3 (5. 6–7. 0)	<0. 0001	7. 4 (6. 4–8. 5)	<0. 0001
Misc*	0. 43 (0. 29–0. 58)	<0. 0001	0. 43 (0. 27–0. 59)	<0. 0001
Business	2. 4 (2. 0–2. 9)	<0. 0001	3. 4 (2. 9–3. 8)	<0. 0001
Holiday	1		1	
Missing				
**Age group (years)**		<0. 0001*		<0. 0001*
0–15	1		1	
16–24	0. 41 (0. 31–0. 58)	0. 002	0. 58 (0. 43–0. 80)	<0. 0001
25–34	0. 20 (0. 16–0. 26)	<0. 0001	0. 26 (0. 19–0. 32)	<0. 0001
35–44	0. 22 (0. 18–0. 31)	<0. 0001	0. 22 (0. 17–0. 28)	<0. 0001
45–54	0. 31 (0. 24–0. 41)	<0. 0001	0. 44 (0. 33–0. 55)	<0. 0001
55–64	0. 36 (0. 26–0. 49)	<0. 0001	0. 55 (0. 41–0. 77)	<0. 0001
65+	0. 49 (0. 33–0. 76)	<0. 0001	0. 45 (0. 31–0. 65)	<0. 0001
Missing				
**Total**				

*Significance test for inclusion of the covariate obtained using likelihood ratio tests. IQR: Inter-quartile range. Misc: Miscellaneous purpose of travel.

Age was independently found to improve the fit of the model (likelihood ratio test *p* < 0.0001, Table 2). All age groups were at lower risk than children (0–15 years old) (Table 2).

When the analysis was repeated using EIR weighted by area, rather that population, the same qualitative patterns were found, with small changes in the quantitative values of the relative hazards (Additional file 4). For example, in the multivariable model the adjusted hazard ratio for travellers visiting friends and relatives was 8.2 (6.4-10.8), and for business travellers it was 3.6 (2.7-4.9). The analysis was also repeated for solely Uganda with different assumptions on travel patterns, where the qualitative patterns were also similar to those across the whole dataset (Additional file 5).

## Conclusions

In 2007, 1,004 cases of *P. falciparum* malaria were reported from amongst approximately 6.5 million UK residents travelling to malaria endemic areas. By comparing the characteristics of these cases with those of all UK travellers to these areas, and adjusting for the variation in exposure between destination countries, these estimates suggest that travellers VFR and, independently, young children are at significantly higher risk of acquiring malaria than other groups. These risks are an aggregate measure of many factors, including chemoprophylaxis, bed net usage and other preventative measures.

Travellers VFR have been identified as a high risk group due to the large number of cases in this group, but previous estimates of enhanced risk have not allowed for an increased EIR in the place that is visited nor their long duration of stay in these areas [3,18,34,35]. The increased risk of acquiring malaria for travellers VFR is likely to be a result of poorer adherence to protective measures, chemoprophylaxis and the perception that areas they stay in are likely to be low risk. Some of these travellers assume that they are immune to malaria due to previous exposure and as a result are less likely to seek pre-travel advice or protect themselves from infection [18,24,36]. Whilst this may indeed be the case for some (perhaps explaining the reduced hazard of acquisition in adults), others may be susceptible. Moreover, recent work highlighted that VFR travellers felt that even if they became infected while staying with friends or relatives, the disease could be dealt with relative ease [37]. This may imply that VFR travellers are less likely to perceive malaria as high risk infection. These results suggest that this group continues to be an important target for prevention messages.

Business travellers also appear to be at increased risk of acquiring infection compared to holiday-makers in both models assessed in this work. Whilst this risk group has not previously been identified in the UK, data from Scotland in 2006–2008 showed that business travellers imported a greater number of cases than VFR travellers despite high reported prophylaxis uptake [16]. Business travellers have also been identified as a risk group in Japan and Switzerland [38,39], and they are a large and increasing group of patients in Europe (almost 19% [1,40,41]), and in the UK the number of business traveller cases has almost doubled between the period 2007–2011 [5]. Even after accounting for duration of stay business travellers were at an increased risk of infection acquisition, this may be because some business travellers avoid protective measures because they travel for short periods or stay in more industrial areas [42] and they are less likely to seek medical advice than tourists [39]. Thus their lack of knowledge, poor awareness and resulting behavior could all contribute to their higher risk.

Imported malaria in children is a complex problem that faces many challenges [43]. The results suggest that children aged 0–15 years could be at a higher risk of infection than other age groups. This is consistent several previous studies [26,44,45]. However, a number of other studies have identified a different pattern of results, which may suggest that young children could be at a reduced risk of acquiring infection relative to other age groups [28,46]. These studies have often focused on children less than five years of age, while this work has assessed a wider age range. Cases in children are of particular concern because of the increased potential for both rapid onset of severe disease, non-specific symptoms and delayed diagnosis in this group [7,28,47,48]. Children of a young age may be at increased risk due to intrinsically higher susceptibility. The use of prophylaxis or personal protective measures remains largely inadequate within this age group, especially amongst those travelling to VFR [28,43]. This altered risk is reflected in case numbers, and should be a priority for public health interventions.

There are a number of limitations of this study. First, it is based on passive surveillance of malaria cases, which is believed to be subject to large underreporting. Second, for some aspects, particularly the reason for travel, there was a large proportion of missing data. Whilst this analysis accounts for these missing data, the mechanism underlying the algorithm used assumes that the data are missing at random. In practice, this may not hold if the reason for travel is systematically not reported by certain groups. Improved recording of this information would be of benefit in defining the groups at highest risk. Equally, data on reason for travel was assumed to be the same across 43 African countries, however there is heterogeneity in reasons for travel to different regions of Africa. Given the limited data available at the country level on purpose of travel it was chosen to keep the proportions available in the TravelPac data. Malaria risk varies substantially within countries. Robert et al. [49] identified a loose linear relationship between mean annual EIR and degree of urbanization. Coupled with this it is also likely that different traveller groups will visit places with variable levels of urbanization, this will affect the force of infection that travellers are exposed to [50]; however the travel destination of cases was only recorded at country level. It was assumed that the distribution of risk which was aggregated according to the population density within each country [11] was the same as the distribution of risk for travellers, which is unlikely to be true for some travel groups. More specific recording of the destination of UK cases at sub-national level would improve estimates of risk [11]. Travel patterns can differ between different reason for travel groups [51], this may result in different forces of infection being experienced by different travel groups at different times of the year as a consequence of different travel preferences at different times of the year, this could result in the seasonal reporting of cases.

Current public health strategies are effectively protecting the majority of UK travellers to malaria endemic areas. However, there are still a number of cases reported each year. This analysis supports previous calls to improve health education and access to prophylactic drugs in immigrant communities in the UK, particularly children. The other group identified here are business travellers, whose perception of the risk of acquiring malaria may be poor, particularly when they are staying for longer than is usual for business travellers. Efforts should, therefore, be made to ensure that prevention messages are reaching this group.

## Competing interests

The authors declare that they have no competing interests.

## Authors’ contributions

TDH, JMR conceived the study, TDH, JMR and JTG designed the analysis. AP and TDH analysed the data. PWG and VS collected and contributed data. All authors wrote and contributed to the manuscript. All authors read and approved the final manuscript.

## Supplementary Material

Additional file 1 **Purpose of Travel data.** Country level data for purpose of travel to different malarial endemic areas. Click here for file

Additional file 2 **The relationship between PfPR2-10 and EIR.** The relationship between PfPR2-10 is illustrated and the details and appropriate citations are provided for the PfPR data, and how it was converted to EIR. Click here for file

Additional file 3 **Statistical methods.** A description of the proportional hazards model developed and an explanation of the expectation maximization algorithm applied to the missing data. Click here for file

Additional file 4 **A sensitivity analysis to the assumptions about EIR within country.** Estimated hazard ratios for the univariable and multivariable models, where the parasite rate across the country was unweighted by population and then converted to EIR. Click here for file

Additional file 5 **Sensitivity to uncertainty in travel patterns – analysis of Uganda.** A sensitivity analysis on uncertainity in travel patterns using Uganda case data. Univariable and multivariable model are presented for estimated hazards using data from the IPS survey and international traveller data. Click here for file
