# Supplementary material for: Risk factors for UK Plasmodium falciparum cases
Source: Malar J. 2014 Aug 4;13:298. doi: 10.1186/1475-2875-13-298 (PMC4132200; doi:10.1186/1475-2875-13-298)
Supplement: Additional file 1 — Purpose of Travel data. Country level data for purpose of travel to different malarial endemic areas. [file 1475-2875-13-298-S1.pdf]

Additional File 1:

Purpose of travel:

As outlined in the main text, data on the purpose of travel and the age of travelers to malaria endemic areas was derived from the TravelPac, provided by the Office for National Statistics [1]. This was grouped at the level of "Other Africa" for 43 of the African malaria endemic countries. In order to investigate the degree of uncertainty associated with these countries, we performed a systematic internet search for data from the governments of each of these countries for data on reason for travel of visitors from the UK. We found only a small number of datasets, and in all cases the proportion of travelers from the whole world, rather than just the UK, with particular reasons for travel were given (see Table S1, below). This highlights a limitation of the current study in using UK traveler behavior summarized across many African countries, but also that more detail is currently extremely difficult to obtain.

Table S1: Additional data collected at the country level assessing purpose of travel to that country.

Country	Date	Notes	Business	Holiday	Misc*	Study	VFR	Other	Source
Other Africa	2007	UK residents. Air travel	13%	45%	6%	1%	35%		Travel pac. International passenger survey
Kenya	2006	All tourists worldwide. Mode of travel not specified.	23%	77%					http://siteresources.worldbank.org/KENYAEXTN/Resources/Tourism_ReportESW_Kenya_Final_May_2010.pdf
Tanzania	2006	All tourists worldwide. Mode of travel not specified.	11%	81%				8%	http://siteresources.worldbank.org/KENYAEXTN/Resources/Tourism_ReportESW_Kenya_Final_May_2010.pdf
Sierra Leone	2007	All tourists. Travel by air. Definition of other not specified.	35%	12%			13%	40%	http://www.theigc.org/sites/default/files/sessions/Cecil%20Williams_Sierra%20Leone_GW2012.pdf
The Gambia	2008	All tourists worldwide. Air travel.	6%	93%			1%	<1%	http://www.visitthegambia.gm/German/images/stories/downloads/monthly_tourist_arrival_statistics_summary_sheet_march_2009.pdf
Uganda	2007	All tourists worldwide. Air travel. Data collected through immigration and emigration forms.	18%	22%			42%	18%	http://www.ubos.org/onlinefiles/uploads/ubos/pdf%20documents/migration2005_09.pdf

*travel to attend sporting events, for shopping, health, religious or other purposes, together with visits for more than one purpose when none predominates (e.g. visits both on business and on holiday).

Reference

1. TravelPac 2007 [http://www.statistics.gov.uk/]
