# Supplementary material for: Risk factors for UK Plasmodium falciparum cases
Source: Malar J. 2014 Aug 4;13:298. doi: 10.1186/1475-2875-13-298 (PMC4132200; doi:10.1186/1475-2875-13-298)
Supplement: Additional file 2 — The relationship between PfPR2-10 and EIR. The relationship between PfPR2-10 is illustrated and the details and appropriate citations are provided for the PfPR data, and how it was converted to EIR. [file 1475-2875-13-298-S2.pdf]

Additional File 2:

Data

Country level EIR:

Data on the proportion of children aged 2-10 with *Plasmodium falciparum* parasites (PfPR2-10) in 2007 was published at a 5km grid scale by Hay et al in 2009 [1]. Subsequent work developed a methodology for summarising this data at the country level by weighting it by the population at the 5km grid scale [2]. For the purposes of this analysis we required an estimate of the country-level entomological inoculation rate (EIR), per year. To do this, the parasite rate (PfPR2-10) was first transformed to EIR using a previously published estimate of the non-linear relationship between these parameters [3], as illustrated in **Figure S1, below**. Once the conversion of PfPR2-10 to EIR had been made at the 5km scale it was converted to the country-level EIR using the population weighting methodology previously described by Gething et al [3].

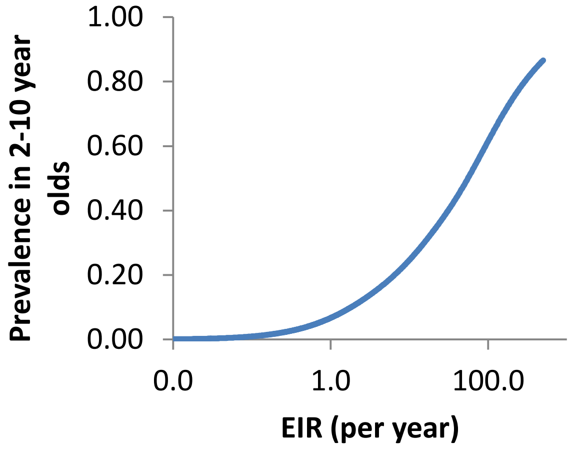

Figure S1: The non-linear relationship between EIR and prevalence of *Plasmodium falciparum* parasites in 2–10 year olds derived by fitting a transmission model to a range of data [3].(Note: In Figure 1 of Griffin et al (2010) a similar relationship is shown between EIR and prevalence in under 15 year olds, which is estimated by fitting the model to a number of datasets giving prevalence in different age groups. This plot is the resulting relationship between EIR and prevalence in 2-10 year olds, for the same parameter values).

References

1. Hay SI, Guerra CA, Gething PW, Patil AP, Tatem AJ, Noor AM, Kabaria CW, Manh BH, Elyazar IR, Brooker S, , Smith DL, Moyeed RA, Snow RW: **A world malaria map: Plasmodium falciparum endemicity in 2007.** *PLoS Med* 2009, **6:**e1000048.
2. Gething PW, Patil AP, Hay SI: **Quantifying aggregated uncertainty in Plasmodium falciparum malaria prevalence and populations at risk via efficient space-time geostatistical joint simulation.** *PLoS Comp Biol* 2010, **6:**e1000724.
3. Griffin JT, Hollingsworth TD, Okell LC, Churcher TS, White M, Hinsley W, Bousema T, Drakeley CJ, Ferguson NM, Basanez MG, Ghani AC: **Reducing Plasmodium falciparum malaria transmission in Africa: a model-based evaluation of intervention strategies.** *PLoS Med* 2010, **7: e1000324**.
