# Supplementary material for: Risk factors for UK Plasmodium falciparum cases
Source: Malar J. 2014 Aug 4;13:298. doi: 10.1186/1475-2875-13-298 (PMC4132200; doi:10.1186/1475-2875-13-298)
Supplement: Additional file 3 — Statistical methods. A description of the proportional hazards model developed and an explanation of the expectation maximization algorithm applied to the missing data. [file 1475-2875-13-298-S3.pdf]

Additional File 3:

Statistical Model

We assume that the risk of acquiring malaria depends on the daily level of exposure (the EIR) and the duration of stay in a malarial endemic country. As such, we assume that the probability of infection per bite is the same for all individuals in all transmission settings. We used a log-linear model to calculate the hazard of acquiring malaria in which the time of infection was assumed to have occurred at any point during the reported visit to a malaria endemic area and the daily hazard of infection is determined by the EIR (assuming a constant rate and thus ignoring the seasonality in transmission since data on the months of visits was not available). Two covariates were considered both in univariable and multivariable models – the age of the participant (as a categorical variable) and the reported reason for travel. The hazard was calculated as an aggregate measure of the effect of the use of preventative measures, such as bed nets, insect repellent and chemoprophylaxis, because detailed usage statistics were not available for travelers in general.

We assume that the risk of acquiring malaria increases with the daily level of exposure in the country visited (the non-seasonal EIR, notated E) and the duration of stay in that country, notated d and use an interval-censored proportional-hazards model for the risk of acquiring malaria for travelers with different purpose of travel and age, i.e. Infection could have occurred at any point during the visit to the malarial endemic area. The baseline hazard of infection was estimated by including the reported travel patterns of all UK residents in the analysis in addition to the reported cases, assuming that any under-reporting of malaria cases would be equally distributed across locations. The probability of acquiring malaria on a visit to a malaria endemic area was given by

$$p = 1 - e^{-\lambda E d}$$

where d is the duration of the visit, E is the population-weighted mean EIR in that country, and λ is the hazard of infection given exposure determined by the log-linear model:

$$\ln \lambda = \alpha + \Sigma_i \beta_i X_i + \Sigma_j \gamma_j Y_j$$

where α is the baseline value, β_i are the coefficients and X_i and Y_i are the covariates (age-group and purpose of travel). The parameters were estimated by maximum likelihood and p-values were calculated using the likelihood ratio test.

Missing data

Since one or more of the covariates or the destination of travel were missing for a proportion of cases (235 (23%) missing information on destination, 455 (45%) missing reason for travel and 4 (<1%) missing age), parameter estimation was performed using an expectation-maximization algorithm [1, 2], the steps of which are as follows:

1. For each item of missing data, the probability that it takes a given value was calculated using the current set of parameters in the model; e.g. for a case where purpose of travel is not known, we use the travel data to give the probability that a traveler to a particular country in a particular age-group would be a traveler VFR, a business traveler or a holiday maker or travelling for miscellaneous reasons. Given these probabilities, we use the model to give the probability that an individual would acquire malaria for each reason for travel in this country. These are then normalized to give the probability that a case arising from a visit to this country would be a particular type of traveler. This probability is then used to calculate the *expected* number of cases with those characteristics under the model.

2. The parameters in the model are then re-estimated by maximizing the likelihood of seeing both the observed (no missing data) and expected (from the cases with missing data) number of cases of each type.

Steps 1-2 are repeated until the parameters converge. For transparency, the analysis was also undertaken excluding the missing data. 95% confidence intervals were obtained from the profile likelihoods. All statistical analysis was conducted using R software [3].

References

1. Do CB, Batzoglou S: **What is the expectation maximization algorithm?** *Nat Biotechnol* 2008, **26:**897-899.
2. Dempster P, A., Laird N, M., Rubin B, D.: **Maximum Likelihood from Incomplete Data via the EM Algorithm.** *Journal of the Royal Statistical Society Series B (Methodological)* 1977, **39:**1-38.
3. R Core Devlopment Team: **R: A Language and Environment for Statistical Computing.** 2005.
