# Supplementary material for: Risk factors for UK Plasmodium falciparum cases
Source: Malar J. 2014 Aug 4;13:298. doi: 10.1186/1475-2875-13-298 (PMC4132200; doi:10.1186/1475-2875-13-298)
Supplement: Additional file 4 — A sensitivity analysis to the assumptions about EIR within country. Estimated hazard ratios for the univariable and multivariable models, where the parasite rate across the country was unweighted by population and then converted to EIR. [file 1475-2875-13-298-S4.pdf]

Additional File 4:

Sensitivity to assumptions about EIR within country

Detailed travel patterns for UK tourists to malaria endemic areas are not available in a systematic way. Given the high degree of local heterogeneity in malaria transmission, it is a limitation of our analysis that we cannot adjust for the local EIR we have performed a sensitivity analysis to our assumptions about travel patterns.

In our baseline analysis (reported in the main paper), we have averaged prevalence across a country by weighting by population density (Additional file 2). This could be interpreted as implicitly assuming that travelers are visiting areas in a country according to their population density. We repeated the analysis under an assumption that people were equally likely to travel to any part of the country, and averaged the parasite rate across the country unweighted by population density (see Table S2, below). The results (Table S2) give a very similar pattern to those presented in the main paper, with a slightly increased adjusted hazard for travelers visiting friends and relatives 8.2 (6.4-10.8) compared to 7.4 (6.4-8.5). This allows some increased confidence that the estimates in the main text reflect a true increase in risk for these travelers when accounting for an estimate of EIR and duration of travel, but does not negate the need for better information on travel patterns to refine the estimates.

Table S2: Estimated hazard ratio (univariable model) and adjusted hazard ratio (multivariable model) for acquiring malaria conditional on the estimated level of exposure in the destination country. This table is similar to the right hand columns Table 1 in the main text, but these estimates are for the calculation of EIR by country (see text above).

	Hazard Ratio (95% confidence interval)	*p*	Adjusted hazard ratio (95% confidence interval)	*p*
**Purpose**		<0.0001 *		<0.0001 *
VFR	6.1 (5.9-7.2)	<0.0001	8.2 (6.4-10.8)	<0.0001
Misc	0.47 (0.45-0.53)	<0.0001	0.47 (0.29-0.57)	<0.0001
Business	2.4 (1.9-3.2)	<0.0001	3.6 (2.7-4.9)	<0.0001
Holiday	1		1	
**Age (years)**		<0.0001 *		<0.0001 *
0-15	1		1	
16-24	0.21 (0.14-0.24)	0.002	0.28 (0.21-0.37)	<0.0001
25-34	0.15 (0.13-0.18)	<0.0001	0.19 (0.15-0.25)	<0.0001
35-44	0.13 (0.10-0.17)	<0.0001	0.12 (0.08-0.15)	<0.0001
45-54	0.30 (0.28-0.34)	<0.0001	0.44 (0.32-0.59)	<0.0001
55-64	0.33 (0.23-0.39)	<0.0001	0.52 (0.38-0.68)	<0.0001
65 & over	0.47 (0.35-0.62)	<0.0001	0.41 (0.32-0.54)	<0.0001

*Significance test for inclusion of the covariate obtained using likelihood ratio tests.
