# Supplementary material for: Risk factors for UK Plasmodium falciparum cases
Source: Malar J. 2014 Aug 4;13:298. doi: 10.1186/1475-2875-13-298 (PMC4132200; doi:10.1186/1475-2875-13-298)
Supplement: Additional file 5 — Sensitivity to uncertainty in travel patterns – analysis of Uganda. A sensitivity analysis on uncertainity in travel patterns using Uganda case data. Univariable and multivariable model are presented for estimated hazards using data from the IPS survey and international traveller data. [file 1475-2875-13-298-S5.pdf]

Additional File 5:

Sensitivity to uncertainty in travel patterns – analysis of Uganda

As discussed in the main text and shown in Additional file 1, there is limited information on reason for travel in many African countries. For a limited number of countries we found estimates of reason for travel from other countries, but none specifically from the UK (Table S1, Additional File 1). Since there were 55 *Plasmodium falciparum* cases in the UK and we had additional data for travel in Uganda (for all travelers, not just UK), we investigated the impact of variation in travel patterns by repeating the analysis in the main text solely for Uganda. Table S3 (below) shows the results for the TravelPac data (line 1 of Table S1, Additional file 1) and Table S4 (below) shows the results for the international data from the Ugandan government website (last line of Table S1, Additional file 1)

Table S3: Characteristics of cases reported amongst travellers to Uganda and estimated hazard ratio (univariable model) and adjusted hazard ratio (multivariable model) for acquiring malaria conditional on the estimated level of exposure in the destination country, using reason for travel data from the IPS

		TravelPac data			All travelers data		
		Adjusted hazard ratio		*p*	Adjusted hazard ratio		
	Cases	(95% confidence interval)			(95% confidence interval)		*p*
**Purpose**				<0.01			
VFR	34	3.28	(1.91 - 7.85)	<0.01	4.16	(2.59 - 40.34)	<0.01
Misc	2	0.1	(0.06 - 0.43)	0.36	0.12	(0.11 - 2.12)	0.32
Business	3	3.21	(0.61 - 9.89)	0.08	4.41	(0.93 - 40.03)	0.07
Holiday	5	1			1		
Missing	11						
**Age (years)**				<0.001			
0-15	8	0.23	(0.24 – 0.45)	<0.01	0.32	(0.15 - 0.52)	<0.01
16-24	9	0.1	(0.18 - 0.39)	<0.001	0.12	(0.06 - 0.37)	<0.001
25-34	13	0.3	(0.4 – 0.83)	<0.01	0.24	(0.14 - 0.59)	<0.001
35-44	13	0.73	(0.62 - 3.54)	0.3	0.64	(0.36 - 1.74)	0.32
45-54	7	1.24	(0.98 - 45.02)	0.61	1.77	(0.87 - 16.89)	0.76
55-64	3	0.99	(0.97 - 72.77)	0.81	0.76	(0.3 - 13.27)	0.46
65 & over	1	1			1		
Missing	1						

Table S4: Characteristics of cases reported amongst travellers to Uganda and estimated hazard ratio (univariable model) and adjusted hazard ratio (multivariable model) for acquiring malaria conditional on the estimated level of exposure in the destination country, using reason for travel data from the national data on international travellers from all countries (see Table S1).

		TravelPac data			All travelers data		
		Adjusted hazard ratio		*p*	Adjusted hazard ratio		
	Cases	(95% confidence interval)			(95% confidence interval)		*p*
**Purpose**				<0.001			<0.0001
VFR	34	3.28	(1.73 - 7.72)	<0.001	4.21	(2.82 – 35.88)	<0.01
Misc	2	0.1	(0.06 - 0.42)	0.36	0.13	(0.11 – 1.72)	0.32
Business	3	3.21	(0.62 – 10.49)	0.08	4.48	(0.95 – 33.59)	0.07
Holiday	5	1			1		
Missing	11						
**Age (years)**				<0.001			
0-15	8	0.22	(0.22 – 0.42)	<0.01	0.32	(0.15 - 0.68)	<0.01
16-24	9	0.1	(0.18 - 0.38)	<0.001	0.12	(0.07 – 0.33)	<0.001
25-34	13	0.29	(0.41 – 0.91)	<0.01	0.24	(0.14 - 0.55)	<0.001
35-44	13	0.71	(0.9 - 3.49)	0.29	0.64	(0.36 - 1.74)	0.32
45-54	7	1.19	(0.94 – 27.56)	0.70	1.78	(0.75 – 11.37)	0.85
55-64	3	0.95	(0.97 – 78.49)	0.80	0.76	(0.3 – 20.28)	0.52
65 & over	1	1			1		
Missing	1						

Since the travel patterns were similar between the data from the IPS and from the national data, the estimates are very similar. This similarity also shows the dominance of the effect of the number of cases from a particular age-group or for a particular reason for travel.
